# Statistical Research on Marine Natural Products Based on Data Obtained between 1985 and 2008

**DOI:** 10.3390/md9040514

**Published:** 2011-03-29

**Authors:** Gu-Ping Hu, Jie Yuan, Li Sun, Zhi-Gang She, Jue-Heng Wu, Xiu-Jian Lan, Xun Zhu, Yong-Cheng Lin, Sheng-Ping Chen

**Affiliations:** 1 School of Chemistry and Chemical Engineering, Sun Yat-Sen University, Xingang Xilu, Guangzhou 510275, China; E-Mails: huguping@mail.sysu.edu.cn (G.-P.H.); cesshzhg@mail.sysu.edu.cn (Z.-G.S.); 2 Zhongshan School of Medicine, Sun Yat-Sen University, Zhongshan Erlu, Guangzhou 510080, China; E-Mails: yuanjie@mail.sysu.edu.cn (J.Y.); sly210@mail.sysu.edu.cn (L.S.); wujh@mail.sysu.edu.cn (J.-H.W.); lanxj@mail.sysu.edu.cn (X.-J.L.); zhuxun333@gmail.com (X.Z.); 3 Key Laboratory of Functional Molecules from Oceanic Microorganisms (Sun Yat-sen University), Department of Education of Guangdong Province, Guangzhou 510080, China

**Keywords:** marine natural products, quantitative analysis, novel compounds

## Abstract

Since the 1960s, more than 20,000 compounds were discovered from marine organisms. In this paper we performed a quantitative analysis for the novel marine natural products reported between 1985 and 2008. The data was extracted mainly from the reviews of Faulkner and Blunt [1–26]. The organisms producing these marine natural products are divided into three major biological classes: marine microorganisms (including phytoplankton), marine algae and marine invertebrate. The marine natural products are divided into seven classes based on their chemical structure: terpenoids, steroids (including steroidal saponins), alkaloids, ethers (including ketals), phenols (including quinones), strigolactones, and peptides. The distribution and the temporal trend of these classes (biological classes and chemical structure classes) were investigated. We hope this article provides a comprehensive perspective on the research of marine natural products.

## 1. Introduction

Marine natural products chemistry was conceived in 1951 when Bergmann and Feeny reported on the isolation of the unusual nucleosides spongouridin and spongothymidin from the sponge *Cryptotethya crypta*, which served as lead structures for antiviral drugs such as Ara-A [27]. More than a decade later, Weinheimer and Spragginsthe discovered prostaglandins in the Caribbean gorgonian Plexaura homomalla at the same time as prostaglandins had just been discovered to be important mediators in the human body, which served as further stimulus in the search for new drugs from the sea [28]. In 1967, a small symposium was held in Rhode Island, USA, with the ambitious title “Drugs from the Sea” [29]. It is the first academic meeting about finding drugs from marine natural products. Although the tone and theme of the meeting was somewhat hesitant, it symbolized marine natural products chemistry’s birth! In general a few novel potential structures for drugs were discovered from marine source between 1950 and 1980. D.J. Faulkner said, “Marine Natural Products Chemistry is essentially a child of the 1970s” [30].

During 1980s the child—Marine Natural Products Chemistry entered adolescence. Novel marine natural products developed rapidly. More and more marine natural products were investigated on a broad spectrum of pharmacological activity.

From 1984, D.J. Faulkner reported a series of annual reviews about marine natural products [1–18]. After 2003, J.W. Blunt and his coworkers took over this work [19–26]. The list of compounds in these reviews is by no means exhaustive, but they do cover the most novel marine natural compounds every year. In this paper, the data mainly from these reviews was quantitatively analyzed. We hope this article may provide a comprehensive perspective in the research of marine natural products.

## 2. Data and Methods

Data used in this paper were obtained from a marine natural products database, which was set up by our research group [31]. The data of this database quote mainly from the reviews of Faulkner and Blunt (we add the data reported before 1980 and published in Chinese). We only selected the compounds which were isolated from marine organisms. From the original paper of every marine natural product, the biological chemical and pharmacological information was abstracted and inputted into the database. Now the database includes a data collection of 12,322 novel compounds isolated from marine organisms. In this paper we performed a quantitative analysis for these marine natural products. Based on their chemical structure, these natural products are divided into seven classes: terpenoids, steroids (including steroidal saponins), alkaloids, ethers (including ketals), phenols (including quinones), strigolactones, and peptides. We also study the organism producing these marine natural products. We used the Faulkner and Blunt’s biological classification to assign organisms to three major biological classes, marine microorganisms (including phytoplankton), marine algae and marine invertebrate. Marine microorganisms (including phytoplankton) included marine actinomycete, marine fungi, marine bacteria and phytoplankton (actinomycete was independently researched due to its unique metabolites). Marine algae include green algae, golden algae, red algae, brown algae and diatoms. Marine invertebrate include protozoa, porifera, cnidarians, bryozoa, molluscs, echinoderms and chordates.

## 3. Results and Analyses

### 3.1. Temporal Distribution

**Figure 1.**
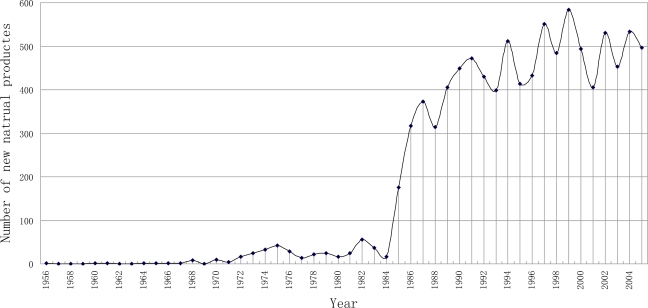
Temporal trend in the number of novel products obtained from marine organisms.

The trends which exhibited a number of novel products obtained from marine organisms are shown in Figure 1; these results are based on the information obtained from the database. Before 1985, natural products discovered annually were less than 100; this number, however, increased to over 300 in 1987, and remained at a constant level of about 500 products per year in the late 1990s. Generally speaking, the marine natural products chemistry has developed rapidly since the mid-1980s. Our data cannot cover the history of marine natural products completely (Faulkner’s reviews began in 1984). However, from other reviews [3–4,32–34] we reached a similar conclusion. The increase in exploitation of marine organisms to obtain novel products has been coincident with the invention and development of the high-resolution nuclear magnetic resonance spectrometer (NMR). In the 1970s, the NMR developed at a high speed, FT-NMR (1970), commercial superconductor magnet (1975) and 2D-NMR (1980) [35] appeared in succession. In fact NMR instruments play a key role in the structure elucidation of marine natural products structure.

### 3.2. Species Distribution

**Figure 2.**
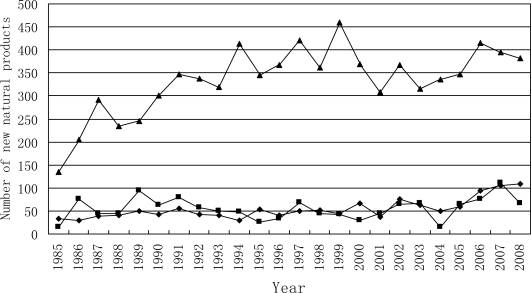
Temporal trends in the number of novel compounds isolated from different marine organisms between 1985 and 2008. ▵, marine invertebrate; ▪, marine algae; ♦, Marine microorganisms (including phytoplankton).

Figure 2 shows the trends in the number of novel compounds isolated from different marine organisms between 1985 and 2008. The number of compounds isolated per year from marine invertebrate increased from 1985 to 1991 and increased steadily to above 350 after 1991. The number of compounds from marine algae changed in a small range, varying between 50 and 100. The number of compounds from marine microorganisms (including phytoplankton) increased slowly from less than 50 in 1980s to over 100 in 2008.

#### 3.2.1. Novel Compounds Isolated from Marine Invertebrates

**Figure 3.**
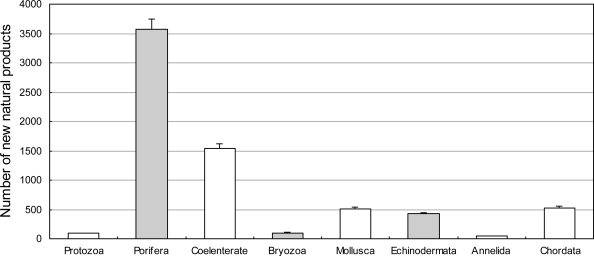
The number of novel compounds isolated from marine invertebrate between 1985 and 2008.

**Figure 4.**
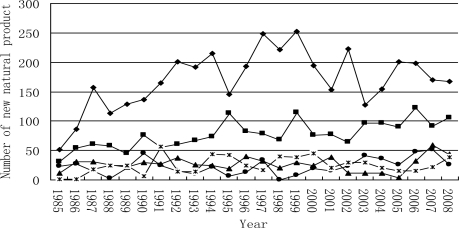
Temporal trends in the number of novel compounds isolated from marine invertebrate between 1985 and 2008. *, Chordates; ▪, cnidarians; ▵, molluscs; ♦, porifera; •, echinoderms.

Until 2008, the majority of novel compounds from marine organisms were isolated from marine invertebrates—approximately 75% of the compounds were isolated from marine invertebrates belonging to the phyla Porifera(mostly sponge) and Coelenterate(mostly coral). This proportion remained between 1985 and 2008 (Figures 3 and 4). Most novel compounds were isolated from sponge and coral, as the sponge is most widely distributed in the world and coral grows in the place with the richest biodiversity, *i.e.*, the tropic ocean. However, it is difficult to cultivate most marine invertebrate on a large-scale. For example, ET-743 is a promising anti-cancer agent yet 1 metric ton (wet weight) of the tunicate E. turbinate has to be harvested and extracted in order to obtain approximately 1 g ET-734 [36]. Therefore, economically viable chemical synthesis techniques are essential for the large-scale development of these products.

#### 3.2.2. Novel Compounds Isolated from Marine Algae

**Figure 5.**
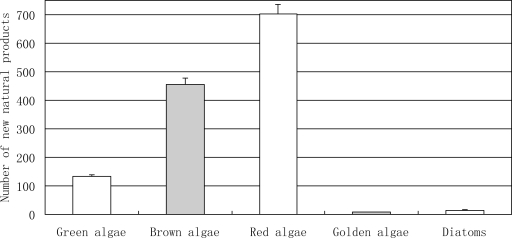
The number of novel compounds isolated from marine algae between 1985 and 2008.

**Figure 6.**
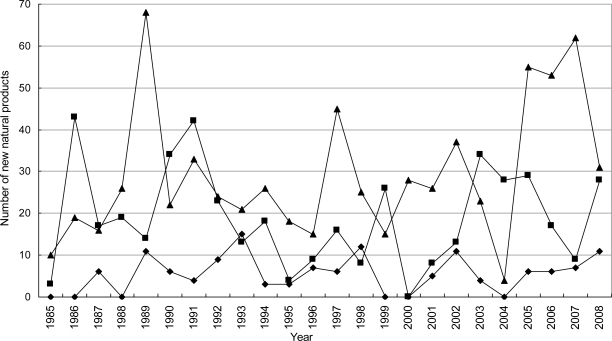
Temporal trends in the number of novel compounds isolated from marine algae between 1985 and 2008. ▪, brown algae; ▵, Red algae; ♦, green algae.

Marine algae became an industrial resource much earlier than marine invertebrate and marine microorganisms (including phytoplankton). This is mainly based on farming of edible species or on the production of agar, carrageenan and alginate. In recent years, pharmaceutical firms have started looking for marine algae in their search for new drugs from marine natural products [37]. According to our statistics, most of the novel compounds were isolated from red algae, brown algae and green algae (Figures 5 and 6). By pharmacological investigation these novel compounds have shown promising biological activities. Most species of red, brown and green algae have been utilized on an industrial scale for one hundred years, which indicates that the novel compounds from marine algae are more suitable as potential drugs than those from marine invertebrate.

#### 3.2.3. Novel Compounds Isolated from Marine Microorganisms (including Phytoplankton)

**Figure 7.**
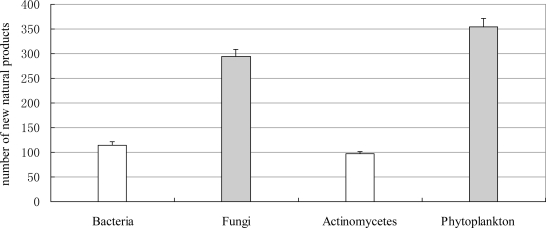
The number of novel compounds isolated from marine microorganisms (including phytoplankton) between 1985 and 2008.

**Figure 8.**
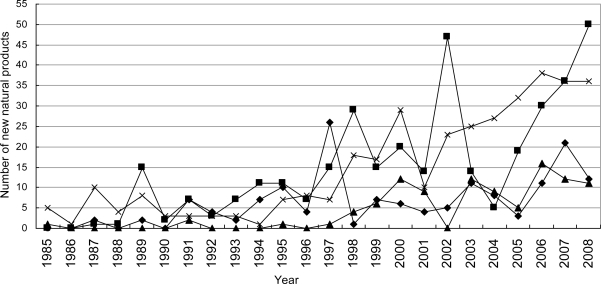
Temporal trends in the number of novel compounds isolated from marine microorganisms (including phytoplankton) between 1985 and 2008. ▪, Fungi; ▵, bacteria; ♦, actinomycetes; ×, Phytoplankton.

Marine microorganism was regarded as one of the potential sources for the discovery of novel structures for new drugs [38,39]. According to our statistics, the number of novel compounds isolated from marine microorganisms (including phytoplankton) was small initially but has been increasing since 1997 (Figures 7 and 8). In the last decade, more and more marine natural product chemists have focused on the marine microorganisms (including phytoplankton). Compared with invertebrate and algae, microorganism multiplies faster and easier to large scale cultivation. In recent years, the investigation of marine microorganisms (including phytoplankton) natural products has been driven forward by microbial genetics. Piel *et al.* answered unsolved questions on the origin of natural products by cloning the pederin biosynthesis gene cluster from the symbiotic bacterium of the *Paederus* beetle [40]. Furthermore, the genome sequencing of the most promising bacterial strains paves the way to discover and mine orphan biosynthetic clusters encoding as yet unexpressed novel compounds. For example, the presence of approximately 39 different biosynthetic loci were revealed in the genome of the marine bacterium *Salinispora arenicola* (GenBank accession No. CP000850) [41]. These biosynthetic loci would be a tremendous source of new chemistry to await discovery and evaluation. The strong interplay between classical natural product chemistry and modern microbial genetics and bioinformatics will therefore help to overcome supply and sustainability issues of the past and to promote marine microorganism natural products as a well-recognized alternative for future drug discovery programs [42].

### 3.3. Chemical Compounds Isolated from Marine Organisms

**Figure 9.**
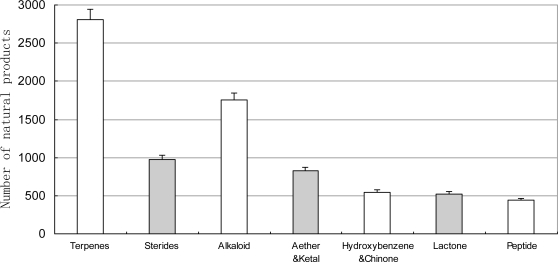
Distribution of the chemical compounds isolated from marine organisms between 1985 and 2008.

**Figure 10.**
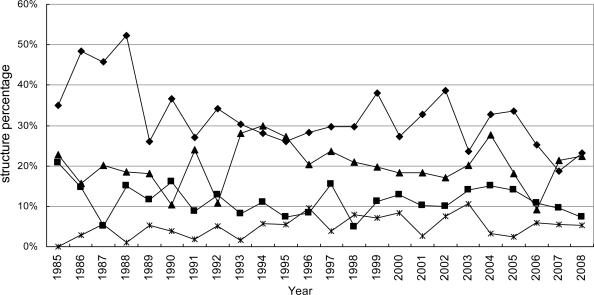
Temporal trends in the percentage of marine natural products reported from 1985 to 2008. *, Peptides; ▪, steroids; ▵, alkaloids; ♦, terpenoids.

**Figure 11.**
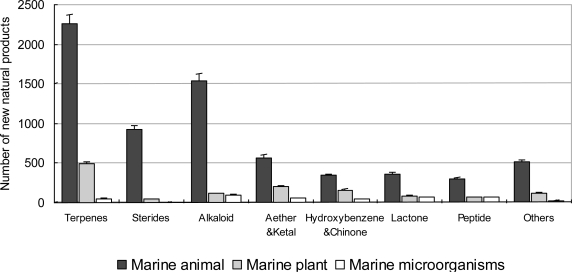
Distribution of novel compounds isolated from marine organisms, between 1985 and 2008.

According to our statistics, the chemical structures are divided into seven classes. The amount of terpenoids and alkaloids was the largest (Figure 9). The amount of all structure classes increased between 1985 and 2008. The proportion of terpenoids decreased from 50% to 30–40% in the late 1980s and then stabilized at that level (Figure 10). Obviously over the last twenty years chemical technology has progressed rapidly, including high resolution MS, high field NMR and high performance LC, so that marine natural products were no longer limited to just a few structure classes. Equipped with a Micro-probe, the High-field FT NMR extended the research of marine natural product to below 1 mg. Chlipala *et al.* researched two new marine polyketide metabolites, both less than 1 mg (0.3 mg, 0.8 mg) [43]. The use of 900 MHz cryoprobe NMR allowed the elucidation of the 2D structure. The cryoprobe probe, capillary probe and Mano probe, were used in the research of marine natural products in smaller amounts [44,45]. This new technology expanded the scope of marine natural products. Among the chemical structure classes, the number of marine invertebrate is the largest (Figure 11). This result is in accordance with Figure 2. There have been no novel sterides isolated from marine microorganisms (including phytoplankton). Our collection is by no means exhaustive so it is credible that a small amount of novel sterides were discovered from marine microorganisms (including phytoplankton). In the decades our research group focused on marine microorganisms, we did not discover any novel sterides. All of the sterides are quite familiar to chemists, for example ergosterin and peroxide ergosterin. We cannot give a reasonable explanation, but we hope this problem can be solved in the future by more investigation.

## 4. Conclusions and Prospects

Unlike terrestrial organisms, marine organisms have to adapt to extreme marine environmental conditions such as high pressure, high salt concentration, low nutrient concentration, low but steady temperature (except the high temperature near underwater volcanoes and the extremely low temperature in polar regions), limited sunlight, and low oxygen content. To acclimatize to these conditions, marine organisms possess unique characteristics that differentiate them from terrestrial organisms in many aspects, such as metabolism, behavior, information transfer, and adaptation strategy. These characteristics are helpful in determining the differences between terrestrial and marine organisms in regards to mechanisms of secondary metabolism and enzyme reactions. These differences are responsible for the diversity in the secondary metabolism of marine organisms.

As discussed in this paper, a broad outlook for marine natural products has been displayed in drug development. The marine natural products isolated from marine invertebrate have a rich novel chemical structure. As large-scale industrial breeding of most marine invertebrate is difficult, most of these structures have been used as a leader structure for chemical or biological synthesis. In contrary to terrestrial organism less novel compounds have been discovered from marine algae (plant) than from marine invertebrate (animal). This is possibly attributable in part to the simpler anatomical structure and reproductive mode of marine algae. Remarkably there are more Multi-halogenated compounds isolated from marine algae; most displaying strong bioactivity. Benefiting from easy industrial culturing and the application of gene technology, marine microorganism (including phytoplankton) is the most promising field in which to develop new drugs. In addition, more and more potential novel compounds isolated from marine invertebrates proved to be biosynthesized by mutualistic microorganisms [46]. These microbes contain biosynthetic genes for use in potential compounds [47]. Tracing and cloning these genes would create effective heterologous producers. Using this metabolic engineering approach opens a new way to develop potential compounds isolated from marine invertebrate.

The vast ocean, which has an area of about 360 million km^2^, possesses incredible resources of novel compounds for investigation by natural product chemists, and marine natural products continue to contribute to novel drug discovery and play a leading role in this field. We believe that the ocean, which hosts approximately 87% of the Earth’s life, offers immense potential for the discovery of pharmaceuticals.
